# An Unusual Presentation of Isolated Pisiform Dislocation: A Case Report of Dorsal and Ulnar Displacement

**DOI:** 10.7759/cureus.105564

**Published:** 2026-03-20

**Authors:** Abdullah B Chandasir, Mitchell J Lomis, Christopher Rogers, Daniel T Childes, Doyle Wallace, Mark Fulcher

**Affiliations:** 1 Orthopedic Surgery, Augusta University Medical College of Georgia, Augusta, USA; 2 Orthopedics, Augusta University Medical College of Georgia, Augusta, USA

**Keywords:** an unusual case, hand injuries, orthopaedic hand surgery, ortho surgery, surgical case reports

## Abstract

Isolated pisiform dislocations are a rarely reported injury. They are typically due to blunt trauma, causing pain and paresthesias in the ulnar side of the wrist. Management options for these injuries are primarily discussed in a limited number of case reports and include closed reduction, open reduction, and pisiformectomy. Of these case reports, the dislocations are described with volar displacement. We present the case of an isolated pisiform dislocation with ulnar and dorsal displacement. This case was managed with closed reduction in the operating room and Guyon’s canal decompression, as well as carpal tunnel release, given ulnar and median nerve paresthesias associated with the injury.

## Introduction

The pisiform is the smallest of the carpal bones and serves as the ulnar-most and most volar structure of the proximal carpal row. Unlike most carpal bones, the pisiform functions as a sesamoid bone embedded within the tendon of the flexor carpi ulnaris, which inserts onto the bone and provides its primary dynamic stabilization. The pisiform serves as an attachment site for multiple soft tissue structures, including the extensor retinaculum, the abductor digiti minimi, and the distal ulnar collateral ligament [1]. In addition, the pisiform articulates with the triquetrum, forming the pisotriquetral joint, which contributes to wrist stability and facilitates force transmission along the ulnar side of the wrist [2]. The pisiform is further stabilized by surrounding ligamentous structures, including the pisotriquetral, pisometacarpal, and pisohamate ligaments. Contraction of the flexor carpi ulnaris produces proximal migration of the pisiform due to its direct tendinous insertion, making it the only muscle that exerts a direct dynamic force on the pisiform [3].

Isolated pisiform dislocations are rare injuries, with approximately 14 cases reported in the literature [4,5]. These injuries most commonly occur as a result of blunt trauma to the volar wrist or forceful contraction of the flexor carpi ulnaris, such as during a fall on an outstretched hand or forceful wrist flexion under heavy tension. Due to the pisiform’s close proximity to neurovascular structures within Guyon's canal, dislocation may result in ulnar nerve compression and associated neurologic symptoms. Previously published literature describes management strategies including closed reduction and splinting, open reduction with suture stabilization, or pisiform excision [3,6,7]. Notably, all previously reported isolated pisiform dislocations have described volar displacement of the pisiform [2,6,8-10].

We report a unique case of an isolated pisiform dislocation with dorsal ulnar displacement that was successfully managed with closed reduction combined with decompression of Guyon's canal and the carpal tunnel.

## Case presentation

A 63-year-old male presented to the emergency room three days after a tree struck the patient's palm while attempting to cut down a tree with a chainsaw. He presented with pain in the left hand with paresthesia in the median and ulnar nerve distributions. Prior to the injury, there was no prior hand trauma or surgery to the left forearm or hand.

**Figure 1:**
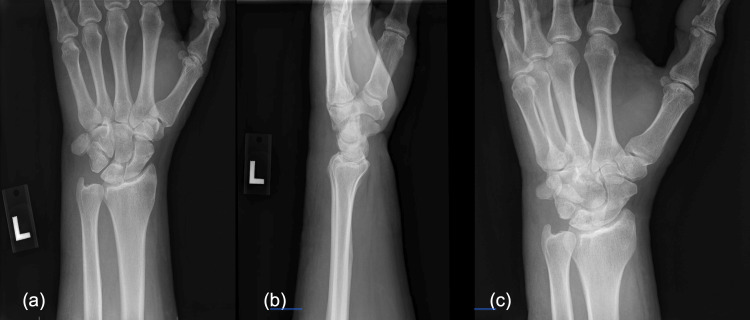
(a) AP, (b) lateral, and (c) oblique radiographs demonstrating dorsal ulnar dislocation of the pisiform without any associated fractures AP: Anteroposterior

**Figure 2:**
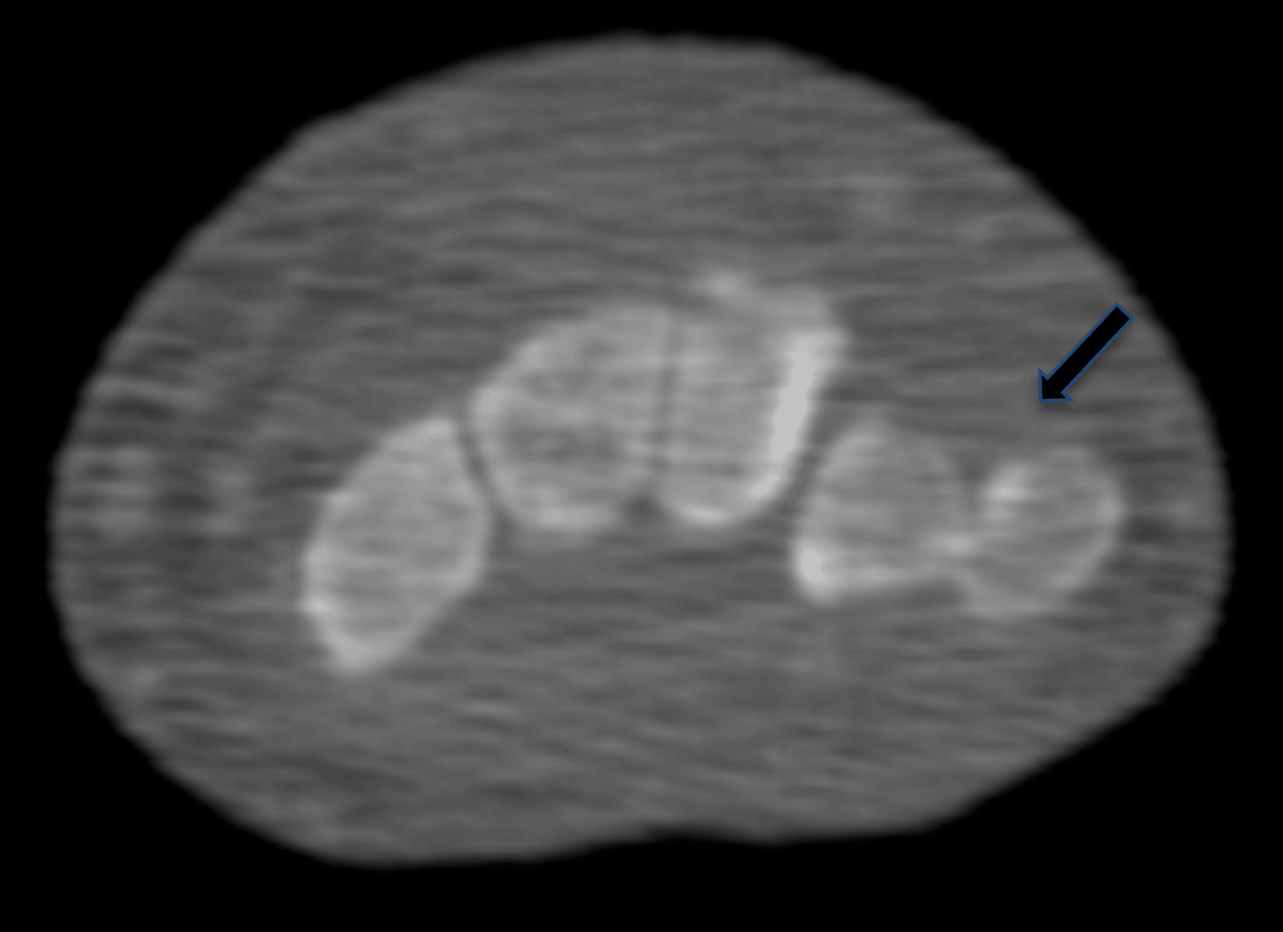
Axial CT demonstrating dorsal ulnar dislocation of the pisiform

**Figure 3:**
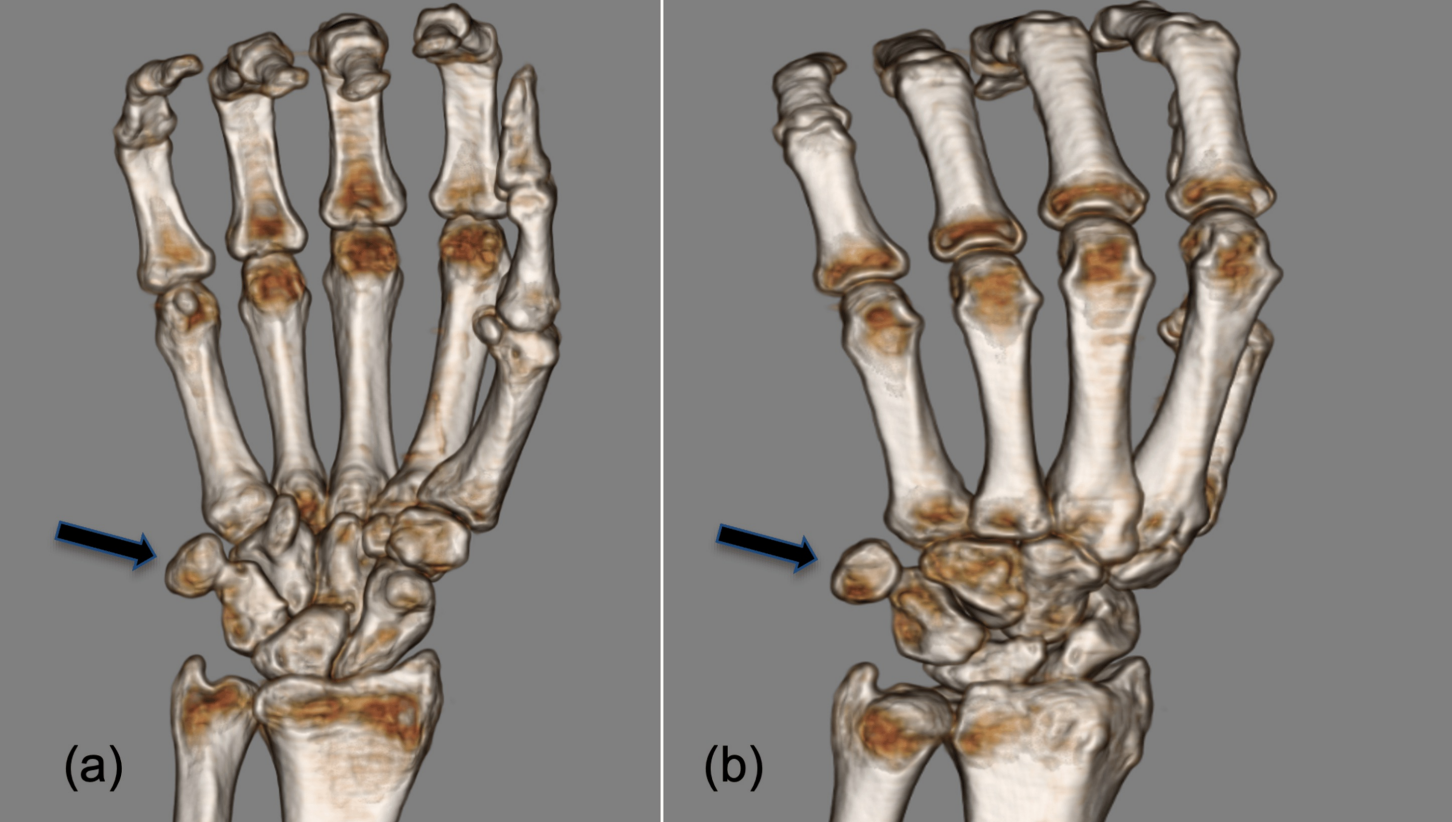
Coronal plane 3D reconstructed images (a, b) of a CT scan displaying dorsal ulnar displacement of the pisiform

On physical exam, the left wrist was noted to be swollen with superficial palmar abrasions. The patient was able to display motor function of the median, ulnar, and posterior interosseous nerves. Sensations within the median and ulnar nerve distributions were subjectively decreased compared to the contralateral side. Radiographs and computed tomography obtained in the emergency department revealed dorsal and ulnar dislocation of the pisiform, shown below (Figures 1-3).

**Figure 4:**
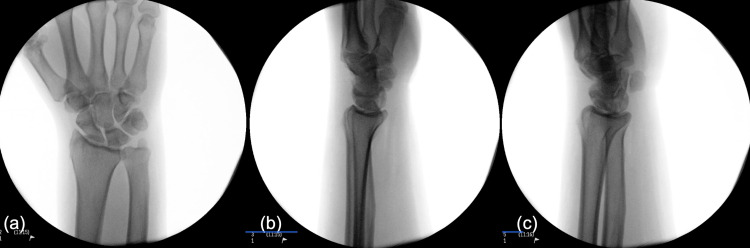
(a) AP, (b) lateral, and (c) oblique intraoperative fluoroscopic images demonstrating reduction of the pisiform AP: Anteroposterior

After extensive discussion, the patient refused closed reduction of the pisiform dislocation in the emergency department. Outpatient follow-up in the hand clinic occurred three days following initial presentation, where he noted continued wrist pain and persistent paresthesias within the median and ulnar nerve distributions. The patient was taken for surgery seven days following the initial injury. At that time, direct manipulation, wrist flexion, and ulnar deviation provided a successful closed reduction of the pisiform. Orthogonal fluoroscopy confirmed appropriate reduction intraoperatively (Figure 4).

The ulnar nerve was decompressed using an extensile approach overlying the left Guyon canal extending across the wrist crease in a Brunner fashion, followed by a left carpal tunnel release. Postoperatively, the patient was immobilized in a wrist cock-up splint. At 13 days postoperatively, the patient continued to report residual paresthesia in his ring finger, but he felt subjective improvement compared with his initial presentation. At six weeks postoperatively, hand and wrist pain had improved markedly; there were no paresthesias within the median and ulnar nerve distributions, and the patient’s splint was removed. He was able to return to all normal activities, and no formal occupational therapy was required. Radiographs displayed maintenance of pisotriquetral reduction.

## Discussion

Compared to other carpal injuries, pisiform dislocations are incredibly uncommon and notably underreported in medical literature. All available literature describes volar dislocations of the pisiform, emphasizing the mechanism of injury, methods of diagnosis, and approaches to treatment for this particular displacement orientation. We present a novel instance in which a patient presented with a dorsal pisiform dislocation.

Given the rarity of pisiform dislocations, there is no uniform consensus on treatment approach. However, several indicators and injury characteristics can guide treatment direction. It is the authors’ belief that the presence of ulnar nerve symptoms helps guide treatment. The average distance of the pisiform to the ulnar nerve is 0.89 ± 0.25 cm [11]. Given the proximity, contusion or entrapment of the ulnar nerve is a real possibility. If paresthesias are present in the ulnar nerve distribution, the surgeon should strongly consider release of the ulnar nerve at the Guyon’s canal. Lin and Hassan reported that Guyon’s canal release with initial pisiform reduction in the OR has provided complete resolution of pain, numbness, and paresthesias postoperatively [8].

**Figure 5:**
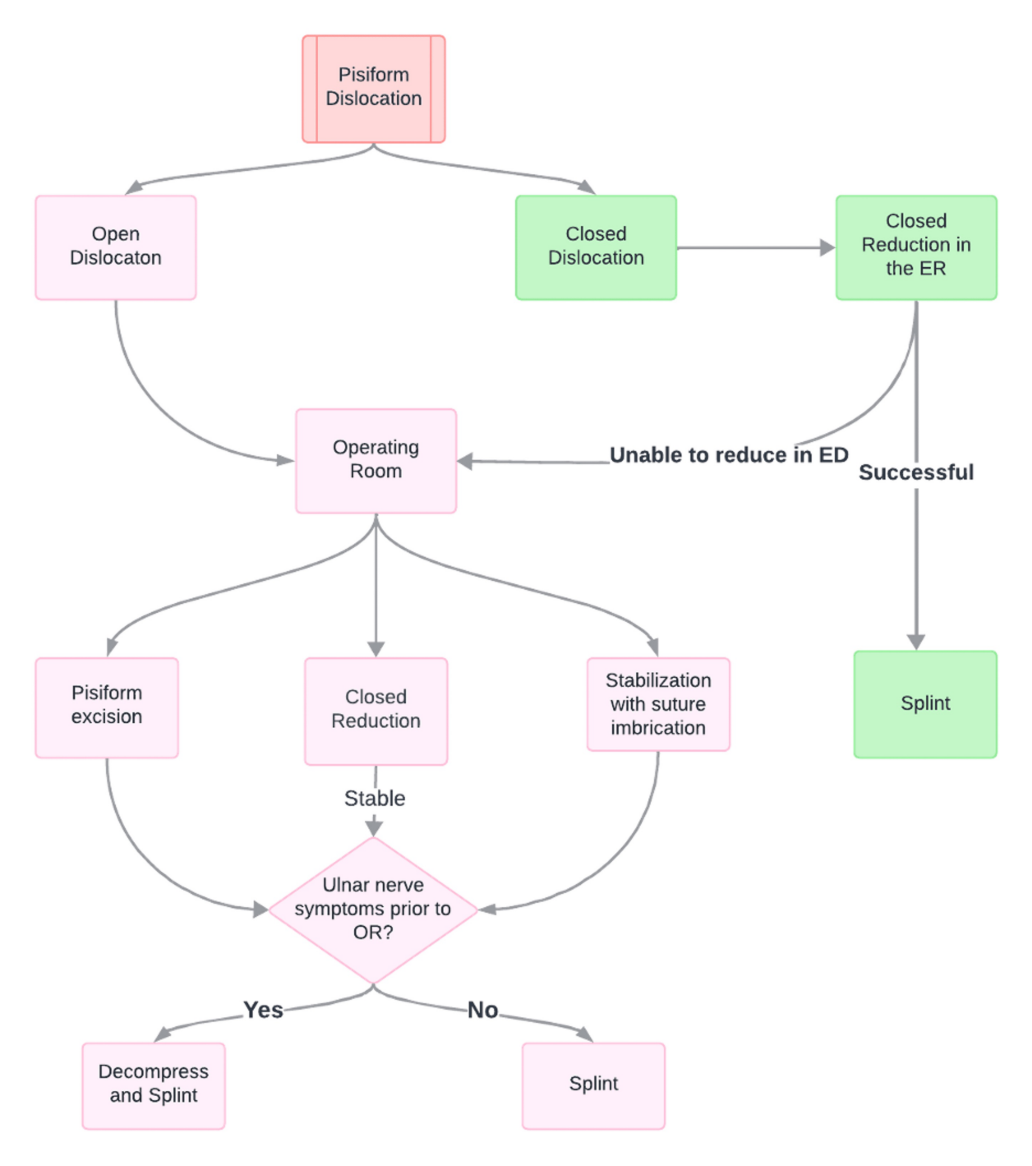
Author-suggested algorithm for the management of pisiform dislocations

When approaching the treatment of pisiform dislocations, an initial closed reduction should be attempted upon presentation. In certain cases, manipulation and closed reduction can yield radiologic reduction and full functional recovery [12]. If a closed reduction in the emergency department is not possible, then the reduction should be attempted in the operating room. In the setting of an irreducible pisiform, open reduction should be performed in the OR. Upon closed or open reduction, Guyon’s canal should be decompressed if ulnar nerve symptoms are present (Figure 5).

Open reduction and internal fixation can be performed with a suture anchor over the distal pole of the pisiform and suture imbrication of the flexor carpi ulnaris tendon [8]. Additionally, excision of the pisiform serves as an alternative, which often yields full functionality and minimal pain [2,13]. Pisiform excision should be favored over stabilization when the diagnosis of the dislocation is significantly delayed or when there are repeat dislocations with the risk of pisotriquetral arthritis [9].

Immediately after pisiform reduction or excision, the patient should have the forearm and hand immobilized in a plaster splint for at least two weeks. Active and passive range-of-motion exercises at the wrist should begin two weeks after surgery [4,6]. Full wrist and hand function is usually regained by two months, at which time full activities can be resumed as tolerated [4,7,9,10]. Patients usually regain full function and return to baseline, with few instances of complications or re-dislocations occurring.

In conclusion, isolated pisiform dislocations are a rare injury and a potential source of ulnar wrist pain in the setting of trauma. In the setting of dorsal and ulnar dislocation with ulnar nerve symptoms, closed reduction with Guyon’s canal release produced a satisfactory functional outcome in this patient. Overall treatment approach should not differ between volar and dorsal dislocations. The authors aim to provide the treating surgeon with a suggested treatment algorithm for this rare injury and demonstrate the unique aspects of dorsal ulnar dislocation (Figure 5).

## Conclusions

Isolated pisiform dislocations are an exceedingly rare clinical entity, with fewer than 15 cases documented in the literature. While historically reported with volar displacement, this case presents a unique dorsal and ulnar displacement pattern resulting from blunt trauma. The injury's proximity to critical neurovascular structures led to acute compression of both the ulnar and median nerves, necessitating a combination of closed reduction and formal decompression of Guyon’s canal and the carpal tunnel. This case underscores the importance of maintaining a high index of suspicion for atypical carpal displacement in the presence of acute paresthesias. Ultimately, prompt surgical intervention and nerve release proved effective in restoring neurological function, adding a novel presentation to the existing body of orthopedic literature.
